# WASH has a critical role in NK cell cytotoxicity through Lck-mediated phosphorylation

**DOI:** 10.1038/cddis.2016.212

**Published:** 2016-07-21

**Authors:** L Huang, P Zhu, P Xia, Z Fan

**Affiliations:** 1 Key Laboratory of Infection and Immunity of CAS, CAS Center for Excellence in Biomacromolecules, Institute of Biophysics, Chinese Academy of Sciences, Beijing 100101, China; 2 Biotherapy Center, The First Affiliated Hospital, Zhengzhou University, Zhengzhou 450052, Henan, China

## Abstract

Natural killer (NK) cells are important effector cells of the innate immune system to kill certain virus-infected and transformed cells. Wiskott–Aldrich Syndrome protein (WASP) and SCAR homolog (WASH) has been identified as a member of WASP family proteins implicated in regulating the cytoskeletal reorganization, yet little is known about its function in lymphocytes. Here we demonstrate that WASH is crucial for NK cell cytotoxicity. WASH was found to colocalize with lytic granules upon NK cell activation. Knockdown of WASH expression substantially inhibited polarization and release of lytic granules to the immune synapse, resulting in the impairment of NK cell cytotoxicity. More importantly, our data also define a previously unappreciated mechanism for WASH function, in which Src family kinase Lck can interact with WASH and induce WASH phosphorylation. Mutation of tyrosine residue Y141, identified here as the major site of WASH phosphorylation, partially blocked WASH tyrosine phosphorylation and NK cell cytotoxicity. Taken together, these observations suggest that WASH has a pivotal role for regulation of NK cell cytotoxicity through Lck-mediated Y141 tyrosine phosphorylation.

Natural killer (NK) cells are the first defense line against viral infections and tumors.^1^ NK cell-mediated lysis of target cells requires the formation of immunological synapse between NK cells and target cells and subsequent delivery of lytic granules containing perforin and granzymes.^2, 3^ The importance of the actin cytoskeleton in this process has been well documented.^4^ However, the precise mechanism of actin reorganization in NK cells remains to be elucidated.

Wiskott–Aldrich syndrome protein (WASP) is the first identified member of an actin regulator family.^5^ WASP family proteins contain a C-terminal domain that binds to and activates the Arp2/3 complex for cytoskeleton remodeling.^6^ In the absence of WASP, cytotoxic activity of NK cells is defective owing to impaired immune synapse formation and perforin localization.^7^ It has also been shown that WASP may be important for integration of NK cell signaling, particularly for nuclear translocation of NFAT2 and NF-*κ*B during the activating receptor NKp46-dependent activation.^8^

WASP and SCAR homolog (WASH) has been discovered as a new WASP family member.^9^ Subsequent studies show that WASH interacts with multiple proteins, including FAM21, to form a large core complex and regulate actin dynamics.^10^ WASH localizes to sorting and recycling endosomes, where WASH complex activates Arp2/3-mediated actin polymerization and controls the production of transport intermediates from endosome.^11^ Unlike other WASP family members, WASH has distinct N-terminal domains, termed WASH homology domain 1 (WHD1) and tubulin-binding region (TBR).^12^ Moreover, WASH has been shown to regulate recycling of many surface receptors via endosomal trafficking in activated T cells.^13^

In our previous works, we found embryonic lethality and extensive autophagy in WASH-deficient mice. WASH recruits RNF2 to ubiquitinate AMBRA1 and inhibits the ubiquitination of Beclin1, a well-known moderator in autophage.^14, 15^ Of interest, WASH is located in cell nucleus and participates in hematopoietic stem cell differentiation through recruiting NURF complex to c-Myc promoter.^16^ However, the role and mechanism of WASH in NK cell function remain poorly understood.

In this study, we show that inhibition of WASH expression with RNA interference or an inducible gene targeting system severely impair NK cell cytotoxicity through blockade of lytic granule polarization. In addition, Src family kinase Lck can interact with and induce tyrosine phosphorylation of WASH protein in human NK cells. These analyses provide the cellular and molecular mechanisms involved in the regulation of WASH function during NK cell activation.

## Results

### WASH has a critical role in NK cell cytotoxicity

**Figure 1:**
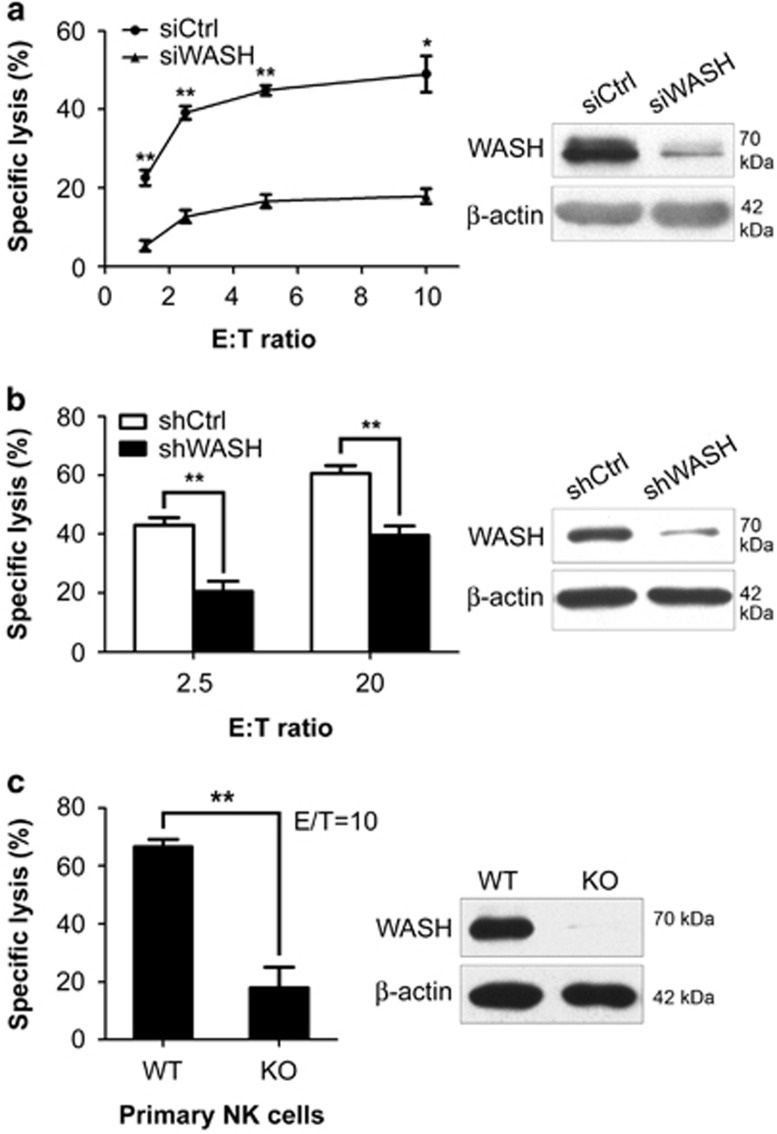
Inhibition of WASH expression impairs NK cell cytotoxicity. (**a**) YTS cells were nucleofected with control siRNA (siCtrl) or WASH-specific siRNA (siWASH) for 48 h. (**b**) YTS cells were stably transduced with control shRNA (shCtrl) or another WASH-specific shRNA (shWASH). Cytotoxic activity of YTS cells were measured against target 721.221 cells in 4-h ^51^Cr-release assays (left panels). A portion of the cells prepared for cytotoxicity was used for analysis of WASH expression by immunoblotting (right panels). (**c**) Primary NK cells were isolated from WT or inducible WASH KO mice. NK cell cytotoxicity against Yac-1 cells was determined by ^51^Cr-release assay at an E/T ratio of 10. All data represent the mean±S.E.M. of triplicates from one representative of three independent experiments (**a**–**c**). Statistical significance was assessed with Student's *t*-test. *P<0.05, **P<0.01

Cytotoxic response is one of the main effector functions by which NK cells quickly remove tumor and infected cells. Cytoskeletal rearrangement is required for the formation of functional immune synapses and the secretion of cytolytic granules. To determine the potential role of WASH in NK cell function, the expression of WASH in human NK cell line YTS was knocked down by RNA interference. First, YTS cells were treated with control or WASH-specific siRNA for 48 h. In a ^51^Cr-release assay, WASH siRNA treatment significantly repressed NK cell cytotoxicity against 721.221 target cells (Figure 1a, left panel). Decreased expression of WASH by siRNA was verified by western blotting analysis (Figure 1a, right panel). Next, we generated YTS cells stably expressing another specific shRNA sequence to inhibit WASH expression. AS shown in Figure 1b (left panel), WASH shRNA-expressing YTS cells also exhibited the reduced capacity to kill 721.221 cells. Lentiviral delivery of WASH-specific shRNA resulted in a remarkable reduction of WASH protein expression compared with control shRNA-treated cells (Figure 1b, right panel). These knockdown results demonstrate that WASH is essential for human NK cell cytotoxicity.

Finally, we confirmed the critical role of WASH in NK cell activity using animal model. Knockout (KO) mice lacking WASH expression are embryonic lethal.^9, 14^ To circumvent this, we recently made use of a conditional loxP-based allele, which enables inducible disruption of WASH by tamoxifen-dependent Cre recombinases (Figure 1c, right panel). Consistent with these human results, NK cell cytotoxicity was defective in fresh NK cells harvested from WASH-deficient mice (Figure 1c, left panel).

### WASH is involved in polarization and degranulation of lytic granules in NK cells

**Figure 2:**
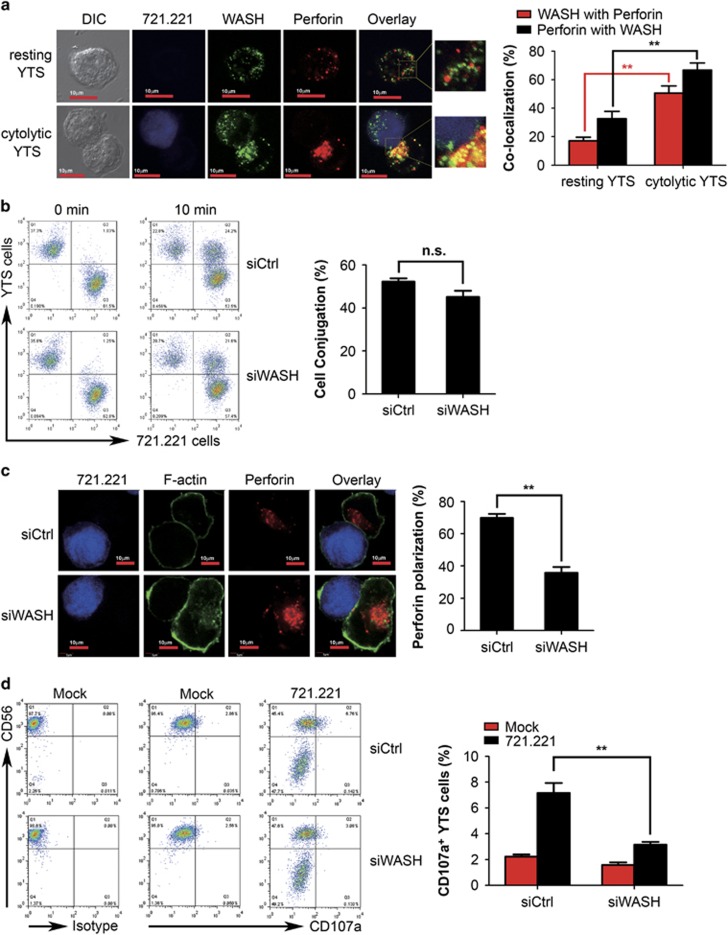
WASH is involved in polarization and degranulation of lytic granules but not conjugate formation. (**a**) WASH associates with lytic granules upon NK cell activation. YTS cells, unconjugated (top panel) or conjugated (bottom panel) by mixing with target 721.221 cells for 15 min at 37 °C, were stained with anti-WASH antibody along with anti-perforin antibody. The graph (right) displays the percentage of colocalization between WASH and perforin. (**b**–**d**) YTS cells were nucleofected with control siRNA (siCtrl) or WASH-specific siRNA (siWASH) for 48 h. (**b**) Conjugate formation between Hoechst blue-labeled YTS cells and CFSE-labeled 721.221 target cells. The graph (right) showed the conjugation percentage within the total YTS cells. (**c**) Perforin polarization to the immune synapse. (**d**) Degranulation of YTS cells stimulated with target 721.221 cells for 6 h in the presence of PE-conjugated anti-CD107a antibody. All data represent mean and S.E.M. from one representative of three independent experiments (**a**–**d**). Statistical significance was assessed with a Student's *t*-test. **P<0.01, NS, no significance

WASH is known to primarily reside at the surface of endosomes in multiple cell lines.^11^ Recently, we discovered that WASH is localized in the cell nucleus in hematopoietic stem cells.^16^ However, information regarding the localization of WASH in well-differentiated lymphocytes, such as human NK cells, is quite limited. To address this issue, we used laser scanning confocal microscopy to visualize WASH expression in both resting and activated NK cells. In the absence of target cells, most NK cells displayed scattered WASH expression, and the overlap between WASH and lytic granules was minimal (Figure 2a, top panel). Upon stimulation with target 721.221 cells, WASH was largely recruited toward the immunological synapse and showed strong colocalization with perforin (Figure 2a, bottom panel), indicating that WASH is recruited to lytic granules during NK cell activation. In addition, the frequency of perforin colocalized with WASH was slightly higher than the frequency of WASH colocalized with perforin, suggesting other locations/functions of WASH in NK cells (Figure 2a). The present experiments suggest that WASH is likely involved in regulation of lytic granule transportation in NK cells.

NK cell-mediated apoptotic killing of target cells comprises several consecutive steps, including target cell adhesion, granule polarization and secretion.^17^ To elucidate the cellular mechanisms of WASH in modulating NK cell cytotoxicity, NK cell effector function was analyzed at each step before lysis of target cells. First, we assessed the role of WASH in cell conjugation between NK cells and target cells. As shown in Figure 2b, knockdown of WASH expression did not significantly affect NK cell conjugation with target cells. Granule polarization is a crucial step in NK cell cytotoxicity.^18^ Next polarization of perforin-containing granules was analyzed in YTS cells stimulated with 721.221 cells. Upon conjugation with 721.221 cells, about 70% of control siRNA-treated YTS cells had perforin granules polarized to the target cell synapse. In contrast, the degree of perforin polarization toward 721.221 cells was remarkably decreased in YTS cells treated with WASH-specific siRNA (Figure 2c). Additionally, siRNA-mediated silencing of WASH expression had no remarkable impact on perforin expression (Figure 2c). Degranulation of cytotoxic lymphocytes can be quantified by determining the surface expression of CD107a in the presence of target cell stimulation.^19^ Finally, we observed that 721.221 cell-induced degranulation was drastically impaired in WASH siRNA-treated YTS cells compared with control siRNA-treated YTS cells (Figure 2d). Taken together, these observations imply that disruption of WASH expression interferes with granule movement and release but not cell contact during recognition of target cells by NK cells.

### WASH is tyrosine phosphorylated in human NK cells

**Figure 3:**
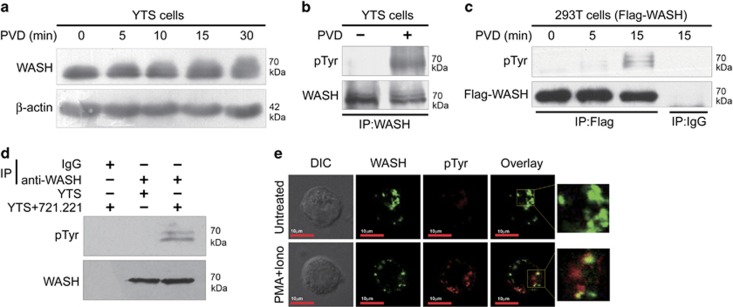
WASH phosphorylation under PVD treatment or NK cell activation. (**a**) YTS cells were stimulated with the phosphatase inhibitor PVD for the indicated time points. Cell lysates were probed with anti-WASH or anti-*β*-actin antibodies. (**b**) YTS cells were treated with PVD for 10 min. IP reactions were carried out with anti-WASH antibody followed by immunoblotting with anti-phosphotyrosine (pTyr) antibody. (**c**) Phosphorylation of exogenous WASH in 293T cells transfected with Flag-tagged WASH and treated with PVD. (**d**) YTS cells were cultured with paraformaldehyde-fixed 721.221 cells at a 1 : 1 ratio for 30 min. Stimulated cells were lysed and immunoprecipitated with control IgG or anti-WASH antibody. Immunoprecipitates were analyzed by immunoblotting with anti-pTyr antibody. All blots are representative of three independent experiments. (**e**) YTS cells were stimulated with phorbol 12-myristate 13-acetate (PMA) and ionomycin (Iono) for 4 h at 37 °C. Cells were then fixed and stained for WASH (green) and pTyr (red). Results are representative of 50 cells

Phosphorylation has been proposed to have a critical role in activation of WASP family members, including WASP and WAVE.^20, 21^ Thus we examined whether protein tyrosine phosphorylation is also an important regulator of WASH activity in NK cells. Treatment of YTS cells with sodium pervanadate (PVD), a powerful inhibitor of protein tyrosine phosphatases, resulted in different migration behavior of WASH protein in SDS-PAGE gel (Figure 3a), suggesting that WASH might undergo phosphorylation followed by PVD treatment. To confirm this observation, we carried out two experiments. First, lysates from PVD-treated YTS cells were immunoprecipitated with anti-WASH antibody and then analyzed by western blotting with anti-phosphotyrosine (anti-pTyr) antibody. The tyrosine phosphorylation of endogenous WASH was observed in human YTS cells after PVD treatment (Figure 3b). Second, 293T cells were transfected with Flag-tagged WASH (Flag-WASH). Cell lysates were immunoprecipitated with anti-Flag antibody and subjected to SDS-PAGE gel. As shown in Figure 3c, Flag-WASH protein immunoprecipitated from PVD-treated 293T cells was also reactive with anti-pTyr antibody.

To determine whether physiological stimulation of YTS cells is associated with WASH phosphorylation, experiments were performed in the presence or absence of paraformaldehyde-treated 721.221 cells. As expected, stimulation of YTS cells by target 721.221 cells resulted in two WASH species detected by anti-pTyr antibody, indicating that WASH is tyrosine phosphorylated under physical activation in human NK cells (Figure 3d). Other kinds of stimuli also induced the accumulation of phosphorylated WASH species in YTS cells (Supplementary Figure S1). Furthermore, confocal microscopy revealed that a small amount of WASH was tyrosine phosphorylated upon stimulation of human NK cell line or primary NK cells with phorbol 12-myristate 13-acetate and ionomycin (Figure 3e and Supplementary Figure S2).

### WASH interacts with Src family kinase Lck

**Figure 4:**
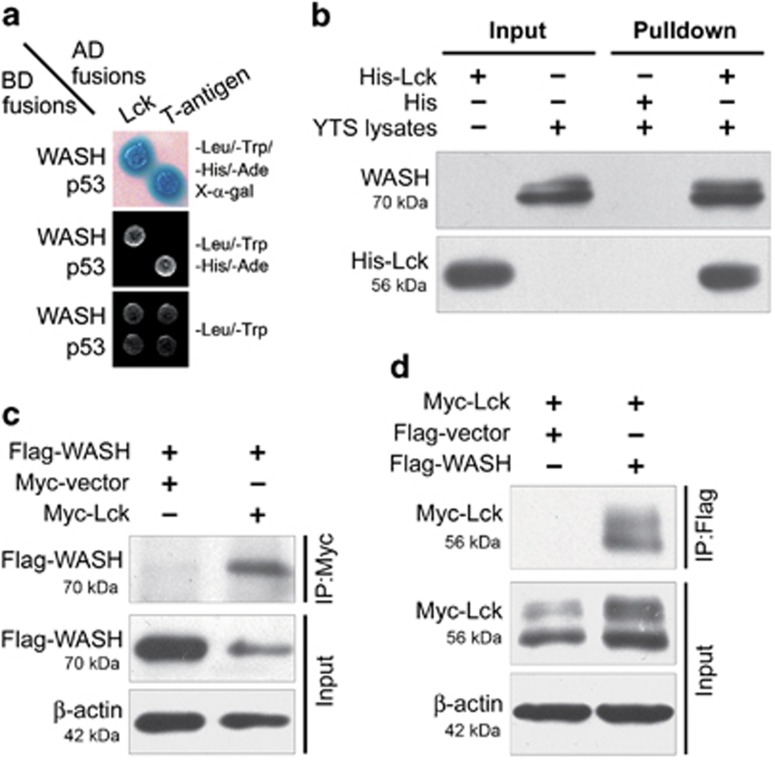
WASH interacts with Src-family kinase Lck. (**a**) Identification of WASH and Lck interaction by yeast two-hybrid assay. Yeast strain AH109 was co-transfected with Gal4 DNA-binding domain (BD) fused WASH and Gal4 activating domain (AD) fused Lck. p53 and large T antigen were used as positive controls. (**b**) His-tagged Lck was expressed in *E. coli* and purified on Nickel-based resin. The YTS cell extracts were incubated with bead-bound His-Lck. Bound WASH was detected by immunoblotting with anti-WASH antibody. 293T cells were co-transfected with Flag-tagged WASH and Myc-tagged Lck for 24 h. Immunoprecipitated proteins were analyzed by immunoblotting with (**c**) anti-Flag or (**d**) anti-Myc antibodies. Data are representative of three independent experiments

To elucidate the molecular mechanism of WASH phosphorylation, we used a yeast two-hybrid system to screen WASH-binding proteins.^14^ One of the candidates is the Src-family kinase Lck, specifically expressed in lymphocytes.^22^ WASH protein interaction with Lck was confirmed by performing additional yeast two-hybrid experiments using the whole WASH protein as bait (Figure 4a). Thereafter, we further analyzed whether WASH interacts with Lck *in vitro* using a pull-down assay. Recombinant His-Lck fusion protein coupled to nickel–agarose beads selectively associated with WASH from YTS cell lysates (Figure 4b), suggesting the interaction between Lck and WASH in human NK cells.

Finally, we confirmed the specific interaction between WASH and Lck in mammalian cells. 293T cells were co-transfected with Flag-tagged WASH and Myc-tagged Lck constructs. Flag-tagged WASH was detected in elutes from the immunoprecipitates with anti-Myc antibody (Figure 4c) and vice versa (Figure 4d). These data strongly implicate that WASH and Lck can physically interact in mammalian cells.

### Src family kinase Lck induces tyrosine phosphorylation of WASH

**Figure 5:**
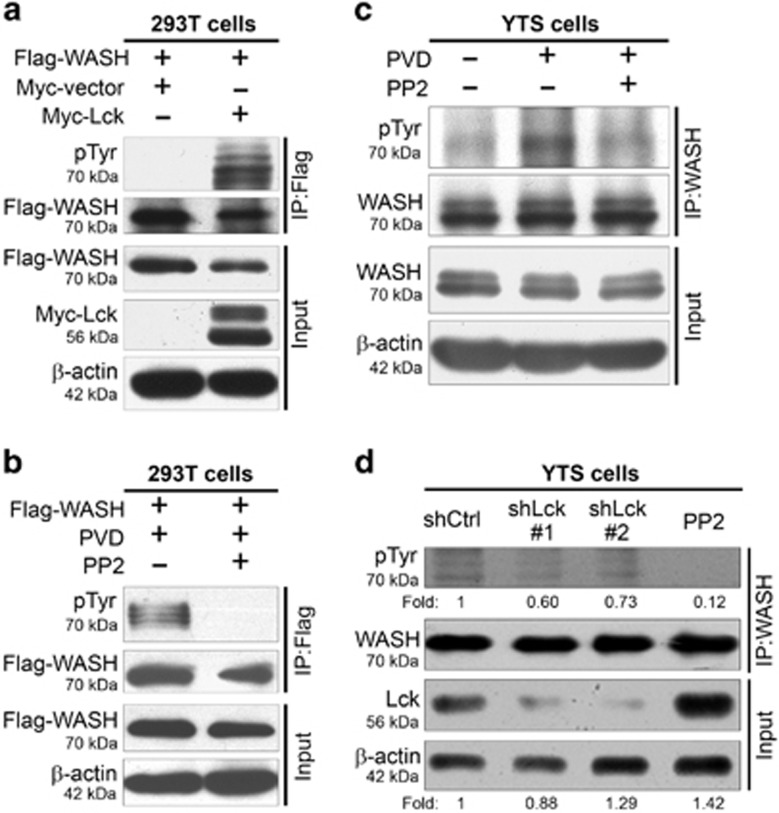
Src-family kinase Lck induces tyrosine phosphorylation of WASH. (**a**) Analysis of WASH phosphorylation in 293T cells co-transfected with Flag-tagged WASH and Myc-tagged Lck. Treatment with a specific Src tyrosine kinase inhibitor PP2 blocked PVD-induced phosphorylation of (**b**) exogenous Flag-WASH in 293T cells and (**c**) endogenous WASH in YTS cells. (**d**) In the presence of paraformaldehyde-fixed 721.221 cells, WASH phosphorylation was partially inhibited in YTS cells transduced with shRNA to specifically target Lck. Data are representative of three independent experiments

The interaction between WASH and the Lck kinase raises the possibility that Lck is relevant to WASH tyrosine phosphorylation. To address the role of Lck in WASH phosphorylation, induction of WASH tyrosine phosphorylation was evaluated in 293T cells overexpressing both Flag-WASH and Myc-Lck. As shown in Figure 5a, Myc-Lck expression efficiently induced tyrosine phosphorylation of Flag-WASH. This result suggests that exogenous expression of Myc-tagged Lck kinase is able to phosphorylate WASH.

To confirm that WASH phosphorylation was mediated by Src family kinase, 293T cells were transfected with Flag-WASH plasmid and incubated in the presence or absence of a Src family inhibitor PP2 before PVD stimulation. WASH phosphorylation was detected using anti-pTyr antibody after immunoprecipitation (IP) of Flag-WASH with anti-Flag antibody. PVD stimulation resulted in significant tyrosine phosphorylation of Flag-WASH, whereas inhibition of Src family kinases completely blocked Flag-WASH phosphorylation (Figure 5b). A similar experiment was conducted in YTS cells to examine phosphorylation of endogenous WASH in human NK cells. Consistently, PP2 treatment attenuated PVD-induced WASH phosphorylation in YTS cells (Figure 5c).

Finally, we knocked down Lck expression by shRNA in YTS cells to confirm the role of Lck in WASH phosphorylation. RNA interference could efficiently decrease the expression level of Lck kinase, whereas PP2 (a pan inhibitor for Src kinases) inhibited protein phosphorylation through blocking the addition of a phosphate group to substrate proteins. As shown in Figure 5d, WASH phosphorylation was significantly suppressed but not completely blocked in Lck shRNA-transduced YTS cells following stimulation with paraformaldehyde-treated 721.221 cells. These data suggest that WASH could be phosphorylated by other Src family kinases besides Lck in human NK cells.

### Phosphorylation of WASH regulates NK cell cytotoxicity

**Figure 6:**
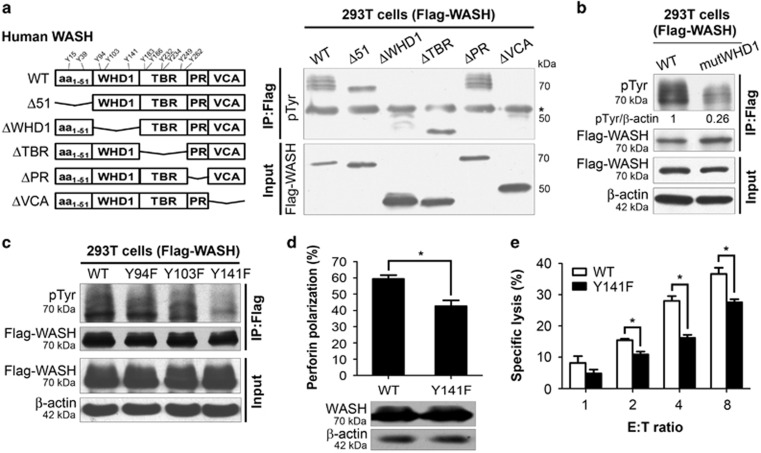
Phosphorylation of Tyr141 in the WHD1 domain of WASH regulates NK cell cytotoxicity. (**a**) Schematic diagrams of WASH functional domains and truncation mutants (left panel). 293T cells were transfected with the Flag-tagged WASH constructs for 24 h followed by PVD treatment for 10 min. Immunoprecipitated proteins using anti-Flag antibody were resolved by SDS-PAGE gel and analyzed by immunoblotting with anti-pTyr antibody (*, non-specific bands). (**b**) Biochemical characterization of the WASH mutant with a Tyrosine-less WHD1 domain (mutWHD1). (**c**) Biochemical analysis of Flag-tagged WT WASH or WASH with each single phosphor-dead mutation (Y to F) in WHD1 domain. (**d**) Perforin polarization in YTS cells expressing WT or mutant WASH (Y141F). (**e**) Specific cytotoxicity of YTS cells expressing WT or mutant WASH (Y141F). Data represent the mean and S.E.M. from one representative of three independent experiments. Statistics were performed using Student's *t*-test (**d** and **e**). *P<0.05

Human WASH has a total of 11 tyrosine residues: Y15, Y39, Y94, Y103, Y141, Y183, Y186, Y232, Y234, Y249, and Y261. Using a panel of truncated WASH constructs, we observed that deletion of aa_1–51_ and TBR domains had no impact on WASH tyrosine phosphorylation but only deletion of WHD1 domain resulted in the loss of PVD-induced WASH phosphorylation (Figure 6a). There is no tyrosine residue in PR and VCA domains. These results implied that the tyrosine candidates for WASH phosphorylation may be located in the WHD1 domain of WASH. Mutation of all three tyrosine resides (Y94F/Y103F/Y141F) in WHD1 domain (mutWHD1) further confirmed the deficiency of WASH phosphorylation in 293T cells transiently transfected with this Flag-tagged mutant WASH (Figure 6b).

We next generated WASH constructs with replacement of each single tyrosine within WHD1 domain. As revealed by anti-pTyr immunoblotting of anti-Flag immunoprecipitates from the transfected cells, induction of WASH phosphorylation was significantly decreased in 293T cells expressing Flag-WASH (Y141F) but was unaffected by mutation at other two WASH tyrosine sites (Figure 6c). These results indicate that Tyr141 is a major site of WASH phosphorylation. To examine the biological relevance of WASH tyrosine phosphorylation in human NK cells, YTS cells were transduced with the WASH mutant (Y141F) and then assayed for NK cell functions. Similar to silencing of WASH expression, mutant WASH (Y141F) expression had little impact on cell growth, as well as apoptosis and proliferation (Supplementary Figure S3 and data not shown). However, both perforin polarization (Figure 6d) and the lysis of target 721.221 cells were considerably reduced in YTS cells expressing mutant WASH (Y141F) compared with YTS cells expressing wild-type (WT) WASH (Figure 6e and Supplementary Figure S4). In summary, these results suggest that tyrosine phosphorylation of WASH has a role in regulating NK cell cytotoxicity.

## Discussion

NK cells require actin reorganization to effectively release cytolytic granules into target cells through the immunological synapse. A central role for WASP in this process has been well characterized through NK cells from WASP patients and animal studies.^7^ In this study, we have demonstrated that the recently identified WASP family member WASH has a significant role in control of NK cell cytotoxicity, indicating that this gene is not redundant with other WASP family members. In addition, Src family kinase Lck can bind to and induce WASH phosphorylation during NK cell activation, providing a potential mechanism to connect WASH regulation with NK cell function. Therefore, it would appear that WASH regulates unique signals required for NK cell activity.

WASH has been implicated in numerous important membrane-trafficking processes, such as endocytosis, vesicular transportation and directional cell migration.^23, 24^ More recently, we found that WASH has a critical role in autophage regulation and hematopoietic stem cell differentiation.^15, 16^ Here we observed a defect in NK cell cytotoxicity in the absence of WASH expression. Our results indicate that WASH regulates polarization and release of lytic granules, a requisite step for NK cell cytotoxicity against susceptible target cells. The cytolytic granules in NK cells are secretory lysosomes undergoing regulated secretion of potent toxins in response to external stimuli.^25^ Our data showed that WASH was recruited to the activating immune synapse in NK cells upon target cell stimulation. In fact, it has been reported that WASH localizes to distinct membrane-enclosed organelles within the cell, such as endosomes, lysosomes and vesicles.^11, 12^ However, inhibition of WASH expression had little impact on NK cells to form conjugates with target cells. In light of these observations, we propose a model for a key role of WASH in NK cell cytotoxicity via regulation of lytic granule trafficking.

We also provide information on the molecular mechanisms involved in regulation of WASH activation. Using a global tyrosine phosphatase inhibitor, we observed that WASH is tyrosine phosphorylated in both endogenous and exogenous expression systems. This observation is consistent with previous reports of WASP activation through tyrosine phosphorylation.^26^ Similar to other WASP family proteins, WASH contains highly conserved regions that are capable of interaction with Src homology 2 (SH2)/SH3-containing tyrosine kinases, such as Src.^27^ Besides its role in actin cytoskeleton remodeling, WASH may also serves as an adaptor protein involved in the downstream signaling induced by contact of NK cells with sensitive target cells.

Lck is a member of Src family kinases and mainly expressed in immune cells. Src family kinases have a key role in many important cellular events, such as cell proliferation, differentiation, survival and cytoskeletal alterations.^28^ Numerous studies have documented that WASP/N-WASP becomes phosphorylated by Src family kinases in response to various stimuli.^26, 29^ However, the molecular mechanism of WASH activation is currently unknown. In the present study, we demonstrated a physiological interaction between Lck and WASH. These observations strongly support the hypothesis that Lck specifically interacts with and phosphorylates WASH to modulate the WASH function in NK cells. In this study, Lck knockdown partially inhibited WASH tyrosine phosphorylation, suggesting that WASH could be phosphorylated by other Src kinases. Whether or not other SH2 domain-containing kinases are responsible for WASH phosphorylation remains to be determined. An interesting finding revealed by this study is that overexpression of Lck protein alters WASH stability or subcellular localization (Figures 4c and 5a). Thus Lck would be expected to promote WASH distribution to the cytoskeleton as it would link signals for dynamic actin remodeling. Further studies are needed to clarify the involvement of cytoskeleton in WASH-regulated NK cell cytotoxicity.

The data reported here identified WASH phosphorylation at Y141 as a major mechanism for induction of WASH activity in NK cells. The association of WASH phosphorylation with NK cell cytotoxicity raises a possibility that phosphorylation of WASH may act upstream of actin filament organization to modulate exocytosis of lytic granules. Phosphorylation of WASP at a conserved tyrosine residue has been shown to positively regulate NK cell activity.^30^ A recent work describes the importance of WASH in recycling of multiple T-cell receptors.^13^ However, implication of WASH in NK cell functions has not been fully explored. NK cells recognize and kill target cells through activating receptors, such as NKG2D and DNAM-1. There is value to explore the impact of WASH on the signal pathways triggered by these receptors during NK cell activation. NK cells and CD8^+^ T cells share many similar properties in recognition and lysis of target cells. The mechanisms of WASH phosphorylation in NK cells or other lymphocytes remains to be further clarified.

In summary, our data suggest that WASH may have a key role in NK cell activity through Lck-mediated phosphorylation. As WASH is also expressed in other immune cells, it may provide a similar function in these cell types. It will also be of great interest to determine the interplay between phosphorylation and other cellular regulations of WASH functions.

## Materials and Methods

### Cell lines, constructs, antibodies and reagents

Human NK cell line YTS cells and human MHC class I-negative B cell line 721.221 cells (provided by Dr. Baoxue Ge, Institute of Health Science, CAS, Shanghai, China) were cultured in RPMI 1640 medium supplemented with 10% fetal bovine serum (FBS), 100 U/ml penicillin and 100 U/ml streptomycin. Human embryonic kidney epithelial 293T (HEK 293T) cells were maintained in DMEM medium, supplemented with 10% FBS and 100 units/ml penicillin and streptomycin. To detect WASH phosphorylation in YTS cells, 721.221 target cells were fixed with 1% paraformaldehyde in PBS for 5 min at 37 °C to inactivate endogenous phosphorylation signals before co-culture with YTS cells.^31^ All constructs were generated by standard PCR to manipulate human WASH gene. For transient overexpression in 293T cells, human WASH and Lck were subcloned into pFlag-cmv-2 vectors and pcDNA4 vector, respectively. For stable overexpression in YTS cells, human WT or mutant (Y141F) WASH were subcloned into pSIN-EF2-IRES-EGFP lentivirus vectors. Commercial available antibodies were mouse anti-human perforin (BD Biosciences, San Diego, CA, USA), mouse anti-*β*-actin, mouse anti-Flag (Sigma, St. Louis, MO, USA), mouse anti-Myc, mouse anti-His (Santa Cruz, Dallas, TX, USA), PE-conjugated CD107a (BD Biosciences), phosphor-Tyrosine mouse antibody pTyr (Cell Signaling, Danvers, MA, USA), Alexa488-conjugated goat anti-rat IgG (BioLegend, San Diego, CA, USA), Alexa488-conjugated donkey anti-mouse IgG, Alexa594-conjugated donkey anti-mouse IgG, Alexa594-conjugated donkey anti-rabbit IgG (Molecular Probes, Carlsbad, CA, USA) and HRP-conjugated secondary antibodies (Santa Cruz). Anti-WASH polyclonal rabbit antibody was generated by our laboratory.

### Knockdown of WASH or Lck in YTS cells

For siRNA-mediated transient transfection, scramble control siRNA and WASH siRNA (5′-GTCCCAGAGAACTACTTCTTT-3′ (sense), 5′–AGAAGTAGTTCTCTGGGACT-3′ (antisense)) were designed and composed in Genepharma Company (Shanghai, China). YTS cells were treated with each siRNA (300 nM) through electric transfection using the Amaxa Nucleofection Kit R (Lonza, Cologne, Germany) by Amaxa program ‘O-017', according to the manufacturer's protocols. The knockdown efficiency was analyzed by western blotting. For shRNA-mediated stable transfection, another RNA sequence against human WASH or two independent Lck sequences were cloned into pSIN-EF2-IRES-EGFP vectors according to standard protocol. For production of lentivirus, 293T cells were bedded onto a 10-cm dish and transfected with 4 *μ*g of pSIN-EF2-shRNA, 4 *μ*g of gag-pol and 2 *μ*g of envelop plasmids using Lipofectamine 2000 (Invitrogen, Carlsbad, CA, USA). Two days later, culture supernatants were harvested and filtered through 0.45 *μ*M filter, and the lentivirus was precipitated by high speed centrifuge (19 500 r.p.m., 2 h) or incubation with PEG-it Virus Precipitation Solution (System Biosciences, Mountain View, CA, USA) for 2 days. YTS cells were infected by the concentrated lentivirus in the presence of 8 *μ*g/ml polybrene. After 2 days of culture, stably infected cells with GFP expression were selected by FACSAria II flow cytometer (BD Biosciences).

### Western blotting

Cell lysate or IP was subjected to SDS-PAGE gel. After electrophoretic separation, the protein was transferred to nitrate cellulose membrane. The membrane was blotted with 5% milk for 2 h and then incubated with primary antibody for 2 h at room temperature or overnight at 4 °C. After washing three times with TBST (130 mM NaCl, 20 mM Tris, 0.1% Tween 20, pH 7.6, 5 min per time), the nitrate cellulose membrane was blotted with HRP-conjugated secondary antibody for 1 h at room temperature and finally visualized by HRP substrates. In some cases, quantification of band intensity was performed using the free ImageJ software (NIH, Bethesda, MD, USA).

### Chromium-51 (^51^Cr)-release assay

For cytotoxicity assay, 721.221 cells were incubated in RPMI1640 supplemented with proper amount of sodium chromate (Na_2_^51^CrO_4_) (PerkinElmer, Boston, MA, USA) for 1 h at 37 °C. After seeding Cr^51^-labeled target cells into 96-well plates (10^4^ cells per well), effector NK cells were added at the indicated concentrations and the plate was incubated for 4 h at 37 °C. Then cells were removed by centrifuge and the radioactivity in supernatants was measured with a gamma counter. Specific lysis ratio (%) was calculated by the standard formula: (experimental−spontaneous release)/(total−spontaneous release) × 100%.

### Confocal microscopy

Cultured YTS cells (10^5^) were washed three times with PBS and mixed with CellTracker Blue prestained 721.221 cells (2 × 10^5^) at 37 °C for 15 min, and then the mixed cells were spread onto poly-L-lysine (Sigma) coated glass slides for 5 min. The slides were rinsed twice to remove non-adherent cells. Cells adhered to the slide were fixed with 4% paraformaldehyde for 20 min and permeabilized with digitonin for 20 min at room temperature. After blocking with 10% donkey serum (Gibco, Carlsbad, CA, USA) for 30 min, cells were stained with primary antibodies at 4 °C overnight. Then the samples were washed with PBS for three times and incubated with proper secondary antibodies. Images were acquired with an Olympus FV1000 laser scanning confocal microscope (Olympus, Tokyo, Japan).

### Cell conjugation assay

For cell conjugation assay, YTS cells (10^5^) and target 721.221 cells (2 × 10^5^) were labeled for 30 min with Hoechst blue and CFSE, respectively, and then mixed together at a ratio of 1 : 2 at 37 °C for 15 min. Finally the samples were detected by flow cytometer, and the conjugation ratio (%) was evaluated by the formula: double positive/Hoechst positive × 100%.

### CD107a releasing assay

For CD107a releasing assay, target 721.221 cells (10^4^) and YTS cells (10^4^) were seeded in 96-well plates and mixed and supplemented with PE-conjugated CD107a antibody. One hour later, monensin was added. After incubation at 37 °C for 4 h, samples were stained with FITC-conjugated CD56 antibody for 30 min and then detected by flow cytometer.

### Immunoprecipitation

Cells were harvested and washed three times with PBS and then treated with lysis buffer (150 mM NaCl, 50 mM Tris-HCl, 1 mM EDTA, 1% NP40, 10 *μ*M Na_3_VO_4_ and protease inhibitor cocktail, pH 7.4) for 30 min at 4 °C. Precipitates were removed from cell lysate by high-speed centrifuge, and the supernatant was precleared by protein A/G beads (Santa Cruz). Two hours later, precleared protein A/G beads were removed. Proper antibody was added for 4 h incubation, and new protein A/G beads were added for 2 h incubation. Next protein A/G beads were collected and washed by centrifuge. Finally, the samples were subjected to SDS-PAGE gel and detected by western blotting.

### Protein expression and purification

The human Lck was cloned into pACYC-duet vector. pACYC-Lck was transformed into BL21 competent cells. After isopropyl *β*-D-thiogalactoside (Sigma) induction at 16 °C for 16 h, *E. coli* were collected and suspended in lysis buffer (50 mM Tris, 500 mM NaCl, 5 mM MgCl2, 1% Triton X-100, 5 *μ*g/ml DNase, 5 *μ*g/ml RNase and 0.25 mg/ml Lysozyme, pH 8.0) and crushed with ultrasonic crusher. Then the lysis supernatant was incubated with His-NTA beads (Invitrogen). His-Lck was recovered with the elute buffer (20 mM Tris, 500 mM NaCl, 500 mM imidazole, pH 7.9) after rinsing with a large amount of washing buffer (20 mM Tris, 500 mM NaCl, 60 mM imidazole, pH 7.9). Elution was dialyzed by the dialysis buffer (20 mM Tris, pH 7.9) and concentrated by high-speed centrifuge.

### Pull-down assay

For Ni-NTA pull-down assay, 30 *μ*l of His resin was charged by Ni^2+^, balanced with the binding buffer and incubated with 200 ng His-Lck in dialysis buffer for 2 h. Then His-Lck-attached Ni-NTA His beads were incubated with YTS cell lysate at 4 °C for 2 h. After washing with the binding buffer for three times, the beads were resolved in loading buffer and boiled for 10 min. The combined proteins were detected by western blotting.

### Yeast two-hybrid assay

AH109 yeast strain was co-transformed with the indicated constructs (pGBKT7-WASH, pGADT7-Lck; pGADT7-SV40 large T-antigen and pGBKT7-murine p53) according to the standard guidelines in the manufacturer's instructions (Clontech, Mountain View, CA, USA). Double transformants were selected with medium lacking leucine and tryptophan. Interaction of fusion proteins was monitored by spotting the selected transformants on plates lacking adenine, histidine, tryptophan and leucine.

### Statistical analysis

Data are presented as mean±S.E.M. and analyzed by Student's *t-*tests (two-tailed). Statistical significance was considered at *P*<0.05.

## Abbreviations

FBS: fetal bovine serum
IP: immunoprecipitation
KO: knockout
NK: natural killer
PVD: pervanadate
SH: Src homology
TBR: tubulin-binding region
WASH: Wiskott–Aldrich syndrome protein and SCAR homolog
WASP: Wiskott–Aldrich syndrome protein
WHD: WASH homology domain
WT: wild type
