# Supplementary material for: WASH has a critical role in NK cell cytotoxicity through Lck-mediated phosphorylation
Source: Cell Death Dis. 2016 Jul 21;7(7):e2301–. doi: 10.1038/cddis.2016.212 (PMC4973352; doi:10.1038/cddis.2016.212)
Supplement: Supplementary Information [file cddis2016212x1.docx]

**Supplementary Information**

Supplementary Figures 1-4

Supplementary Figure Legends

**Supplementary Figure 1** WASH phosphorylation in activated YTS cells. YTS cells were stimulated with different stimuli, including pervanadate (PVD), lipopolysaccharide (LPS), phorbol 12-myristate 13-acetate (PMA) and ionomycin (Iono). Cell lysates were probed with anti-WASH or anti-β-actin antibodies (*, phosphorylated WASH species). A specific Src tyrosine kinase inhibitor PP2 was used as a control to block tyrosine phosphorylation. Representative data of two independent experiments were shown.

**Supplementary Figure 2** Human primary NK cells were isolated from peripheral blood and stimulated with PMA and Iono for 4 h at 37°C. Cells were then fixed and stained for WASH (green) and pTyr (red) antibodies. Fluorescent images were taken by Olympus FV1000 confocal microscope. Representative data of 30 cells were shown.

**Supplementary Figure 3** Effect of WASH expression on YTS cell proliferation. (a) YTS cells were treated with control siRNA (siCtrl) or WASH-specific siRNA (siWASH) for 48 h. siWASH had little impact on YTS cell proliferation. (b) Cell growth analysis for YTS cells expressing wild-type (WT) or mutant (Y141F) WASH. Expression of mutant (Y141) WASH had little impact on YTS cell proliferation. Results are representatives of three independent assays.

**Supplementary Figure 4** Specific cytotoxicity of YTS cells expressing WT or mutant (Y141) WASH against 721.221 cells at an E/T ratio of 10. YTS cells treated with siCtrl or siWASH were used as controls. Representative data of two independent experiments were shown. Statistical significance was assessed with a student *t*-test. **P*<0.05
