# Supplementary figures and images for: WASH has a critical role in NK cell cytotoxicity through Lck-mediated phosphorylation
Source: Cell Death Dis. 2016 Jul 21;7(7):e2301–. doi: 10.1038/cddis.2016.212 (PMC4973352; doi:10.1038/cddis.2016.212)

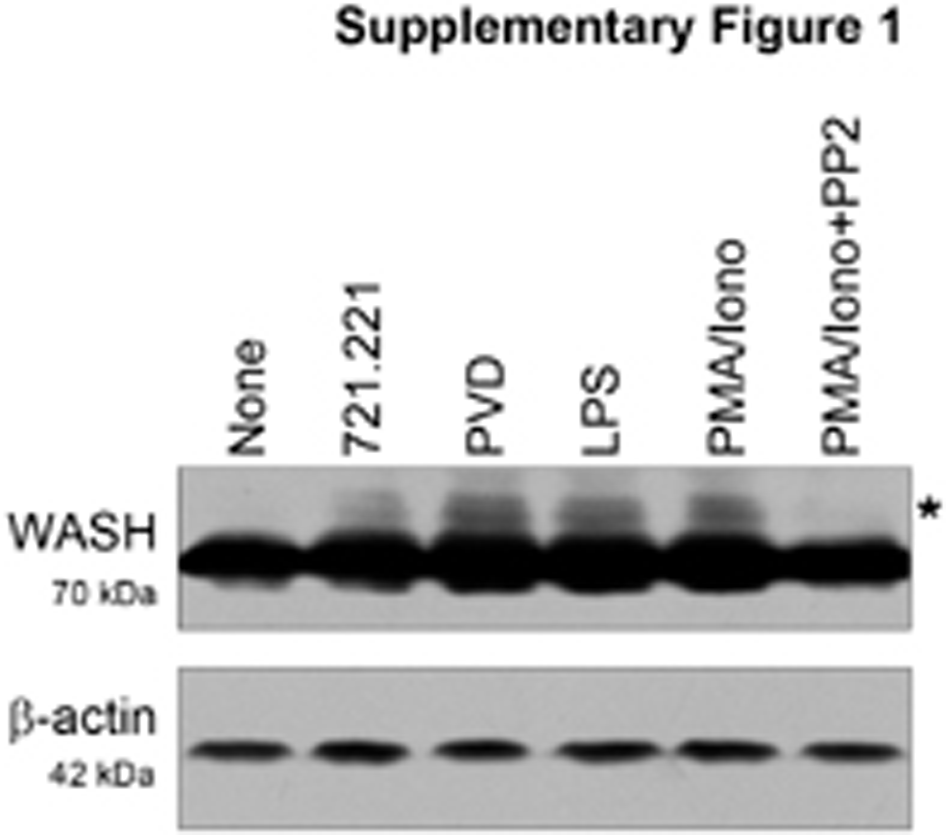

Supplement: Supplementary Figure S1 [file cddis2016212x2.tif]

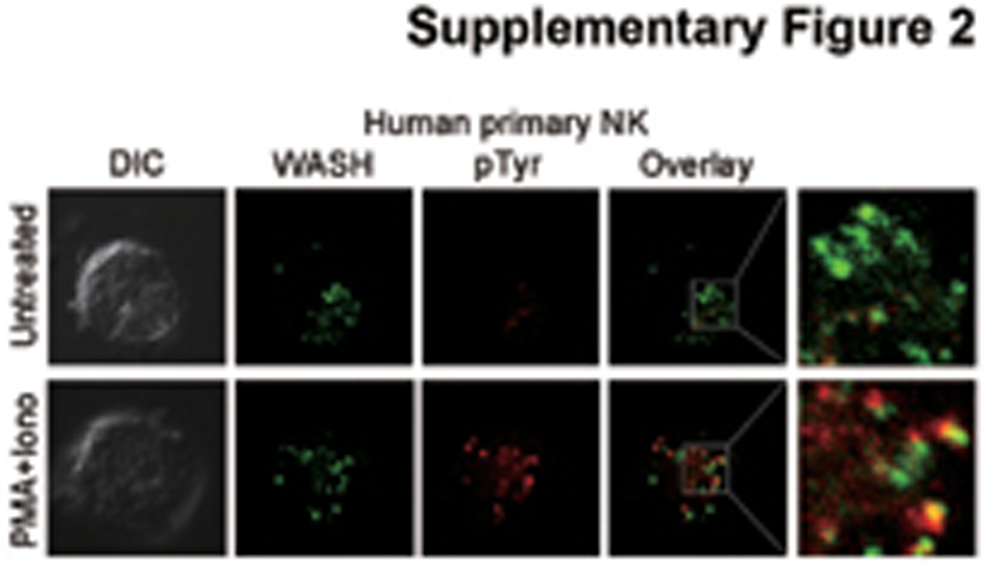

Supplement: Supplementary Figure S2 [file cddis2016212x3.tif]

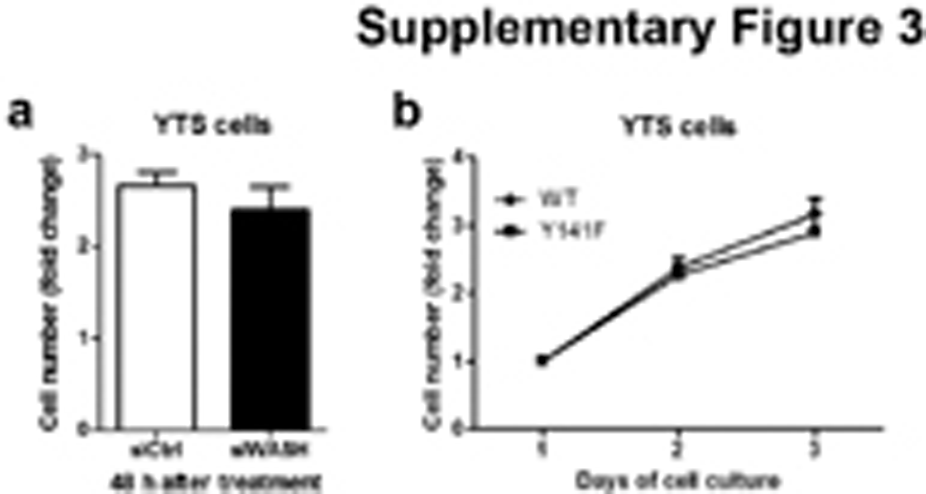

Supplement: Supplementary Figure S3 [file cddis2016212x4.tif]

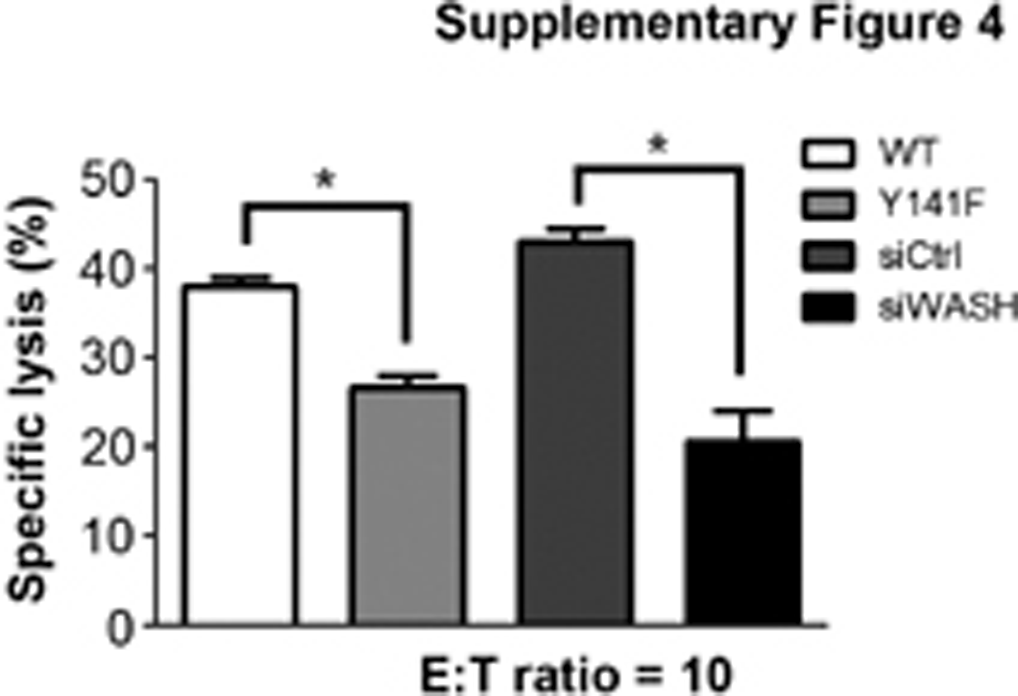

Supplement: Supplementary Figure S4 [file cddis2016212x5.tif]
